# LPS Exposure Increases Maternal Corticosterone Levels, Causes Placental Injury and Increases IL-1Β Levels in Adult Rat Offspring: Relevance to Autism

**DOI:** 10.1371/journal.pone.0082244

**Published:** 2013-12-02

**Authors:** Thiago B. Kirsten, Luciana L. Lippi, Estela Bevilacqua, Maria M. Bernardi

**Affiliations:** 1 Department of Pathology, School of Veterinary Medicine, University of São Paulo, Sao Paulo, São Paulo, Brazil; 2 Department of Cell and Developmental Biology, Institute of Biomedical Sciences, University of São Paulo, Sao Paulo, São Paulo, Brazil; 3 Graduate Program of Environmental and Experimental Pathology and Graduate Program of Dentistry, Paulista University, Sao Paulo, São Paulo, Brazil; Université Libre de Bruxelles, Belgium

## Abstract

Maternal immune activation can induce neuropsychiatric disorders, such as autism and schizophrenia. Previous investigations by our group have shown that prenatal treatment of rats on gestation day 9.5 with lipopolysaccharide (LPS; 100 μg/kg, intraperitoneally), which mimics infections by gram-negative bacteria, induced autism-like behavior in male rats, including impaired communication and socialization and induced repetitive/restricted behavior. However, the behavior of female rats was unchanged. Little is known about how LPS-induced changes in the pregnant dam subsequently affect the developing fetus and the fetal immune system. The present study evaluated the hypothalamic-pituitary-adrenal (HPA) axis activity, the placental tissue and the reproductive performance of pregnant Wistar rats exposed to LPS. In the adult offspring, we evaluated the HPA axis and pro-inflammatory cytokine levels with or without a LPS challenge. LPS exposure increased maternal serum corticosterone levels, injured placental tissue and led to higher post-implantation loss, resulting in fewer live fetuses. The HPA axis was not affected in adult offspring. However, prenatal LPS exposure increased IL-1β serum levels, revealing that prenatal LPS exposure modified the immune response to a LPS challenge in adulthood. Increased IL-1β levels have been reported in several autistic patients. Together with our previous studies, our model induced autistic-like behavioral and immune disturbances in childhood and adulthood, indicating that it is a robust rat model of autism.

## Introduction

Prenatal viral and bacterial infections impair short- and long-term behavior and central nervous system activity in animals [1-3]. Maternal immune activation can also induce neuropsychiatric disorders, including schizophrenia and autism [4-7]. Previous investigations by our group have shown that the prenatal treatment of rats on gestational day (GD) 9.5 with lipopolysaccharide (LPS; 100 μg/kg, intraperitoneally [i.p.]), an endotoxin that mimics infection with gram-negative bacteria, impaired communication and socialization and induced repetitive/restricted behavior in male rats. However, the behavior of female rats was not altered [8,9]. These results suggested that prenatal LPS exposure induced autism-like effects in offspring [9]. In addition to behavioral impairments, our model of prenatal LPS exposure resulted in striatal dopaminergic impairments in offspring, including reduced levels of tyrosine hydroxylase, dopamine and metabolites [9,10]. Moreover, in pregnant rats, LPS exposure induced sickness behavior, including reduced open-field general activity [8].

Although a considerable amount is known about the behavioral alterations and brain damage induced by prenatal LPS exposure, little is known about how maternal immune activation influences the immune systems of animals. Previously, we demonstrated that the immune system can be affected, as evidenced by an attenuated response to experimentally induced asthma [11]. Likewise, little is known about what LPS-induced changes occur in the pregnant dam that subsequently affect the developing fetus. Thus, in the present study, we performed reproductive, neuroendocrine and immune evaluations, both in dams and in their offspring, to better understand what triggered the impairments in the offspring. For this purpose, we evaluated the serum corticosterone levels, the placental tissue and the reproductive parameters in dams that received LPS on GD 9.5. Serum corticosterone and pro-inflammatory cytokine levels were also evaluated in the offspring with or without LPS challenge in adulthood. Because we have already demonstrated that prenatal LPS exposure induces autistic-like effects in infancy [8,9], the present study investigated for persistent changes in adulthood, similar as occurs in autism.

## Materials and Methods

### Ethics Statement

This study was carried out in strict accordance with the recommendations in the Guide for the Care and Use of Laboratory Animals of the National Institutes of Health. The protocol was approved by the Committee on the Ethics of Animal Experiments of the School of Veterinary Medicine, University of São Paulo, Brazil (Permit Number: 1398/2008). All efforts were made to minimize suffering. The experiments were performed in accordance with good laboratory practice protocols and with quality assurance methods.

### Animals

Forty-nine pregnant Wistar rats between 12 and 13 weeks of age and weighing 211–275 g were used. GD 0 was defined as the day when spermatozoa were detected in the vaginal smear. The dams were individually housed in polypropylene cages (38 x 32 x 16 cm) under controlled temperature (22°C ± 2°C) and humidity (65–70%) conditions with artificial lighting (12-hr light/12-hr dark cycle, lights on at 6:00 AM). The animals had free access to Nuvilab rodent chow (Nuvital Co., Sao Paulo, Brazil) and filtered water. Sterilized and residue-free wood shavings were used for animal bedding. The animals were divided into control (saline-treated, SAL, n = 24) and experimental (LPS-treated, n = 25 dams) groups. Different dams were used for the (1) corticosterone (2) reproductive and (3) offspring studies. For the offspring studies, the dams were allowed to give birth and nurture their offspring normally. The day of birth was recorded as postnatal day (PND) 1. No handling was performed on PND 1, but on PND 2, eight offspring (four males and four females) were randomly selected for the following studies. No cross-fostering procedure was used. Litters with fewer than eight pups were culled. The pups remained with each dam until weaning (PND 21). On PND 21, littermates were separated and cohoused by sex under the same conditions as their parents. One male from each litter was used for offspring evaluations to avoid litter effects. The female offspring were separated for use in other experiments. All of the experiments were performed between 9:30 and 10:30 AM to minimize the effects of circadian rhythms. Testing of control and LPS-treated rats was intermixed.

### Treatment

LPS (from Escherichia coli; Sigma, St. Louis, MO; serotype 0127: B8) was dissolved in sterile saline (50 µg/ml LPS in a 0.9% NaCl solution) and administered i.p. to pregnant dams at a dose of 100 µg/kg on GD 9.5. This dose was selected based on our previous findings of maternal sickness behavior and behavioral and brain impairments in offspring [8,10]. The control group consisted of pregnant rats that received only sterile saline (0.9% NaCl), with the same treatment schedule as the LPS-treated animals. Each control dam was treated with a 0.2 ml/100 g saline solution.

### Maternal serum corticosterone determination

Corticosterone is the most abundant circulating steroid secreted by rodents and is considered to be a good indicator of hypothalamic-pituitary-adrenal (HPA) axis activity in these species [12,13]. On GD 9.5, 90 minutes after exposure to LPS (n = 9) or saline (n = 8), dam serum corticosterone levels were determined using commercially available radioimmunoassay kits (Coat-a-Count; DPC, Los Angeles, CA, USA), as previously described [14]. To reduce variability in serum corticosterone levels, rats were handled daily for three days to habituate them to the experimental conditions of euthanasia and blood collection. The animals were euthanized by rapid decapitation.

### Reproductive performance and evaluation of placental tissue

On GD 16, the dams exposed to LPS or saline (n = 6 for each group) were evaluated for reproductive performance and placental tissue. The rats were rapidly decapitated and an exploratory laparotomy was performed with each uterine horn exposed to determine the implantation sites, resorptions and numbers of live and dead fetuses. The placentas were collected for further morphological analysis and the ovaries were removed for corpora lutea counting. The reproductive parameters analyzed were as follows: % pre-implantation loss (number of corpora lutea [CL] – implantation sites [IS] x 100/CL); % post-implantation loss (IS – number of live fetuses x 100/IS; % implantation sites (100 – % pre-implantation loss); and % live fetuses (100 – % post-implantation loss). The placentas were fixed in formaldehyde that was diluted in 10% phosphate-buffered saline (PBS) and then cut into thick slices containing the fully-extended placenta and the fetal and maternal faces. The fragments were placed in cassettes and oriented during the paraffin embedding procedure so that the cut face included a transverse view of the entire placenta. Sections 5-7 µm thick were obtained and stained with hematoxylin-eosin (HE). At least four sections from each placenta were obtained and analyzed using a light microscope.

### Offspring serum corticosterone determination

On PND 60–67, one adult male rat from each experimental and control litter was evaluated for serum corticosterone levels, as previously described in the section *Maternal serum corticosterone determination*. In addition to the prenatal LPS and SAL groups, two other groups were tested for corticosterone levels 90 min after an additional adult LPS challenge (100 µg/kg, i.p.). Thus, there were four groups in this experiment (n = 10 for each group): SAL, prenatal saline injection; LPS, prenatal LPS injection; SAL+LPS, prenatal saline and adult LPS injection; and LPS+LPS, prenatal and adult LPS injection. The LPS challenge dose and time interval have previously been reported to cause sickness behavior and the release of proinﬂammatory cytokines and glucocorticoids [15].

### Offspring serum cytokine determination

It has been suggested that the effects of maternal LPS exposure on the developing fetal brain are mediated by proinflammatory cytokine induction within the maternal circulation and placenta [16,17]. Thus, we evaluated offspring serum concentrations of interleukin-1β (IL-1β) and tumor necrosis factor-α (TNF-α) in duplicate using a commercially available DuoSet^®^ ELISA (Enzyme-Linked Immunosorbent Assay, Development System, R&D Systems, Minneapolis, USA). The blood samples were from the same animals evaluated in the section *Offspring serum corticosterone determination*. There were four groups in this experiment: SAL, LPS, SAL+LPS and LPS+LPS (n = 10 for each group). The methodology used was the same as previously described [18] and was performed according to the manufacturer's instructions.

### Statistical Analyses

The pregnant dam and one male from each litter were the experimental unit. Homoscedasticity was veriﬁed using an F-test or Bartlett’s test. Normality was veriﬁed using the Kolmogorov-Smirnov test. Student’s t-test (unpaired, two-tailed) was used to compare the parametric data of two groups (SAL and LPS). One-way ANOVA followed by Tukey’s test was used to compare the parametric data of four groups (SAL, LPS, SAL+LPS and LPS+LPS). Nonparametric data of two groups (SAL and LPS) was analyzed using the Mann-Whitney U test. The results are expressed as the mean ± SEM or median (minimum and maximum). In all cases, the results were considered signiﬁcant at p < 0.05.

## Results

**Figure 1:**
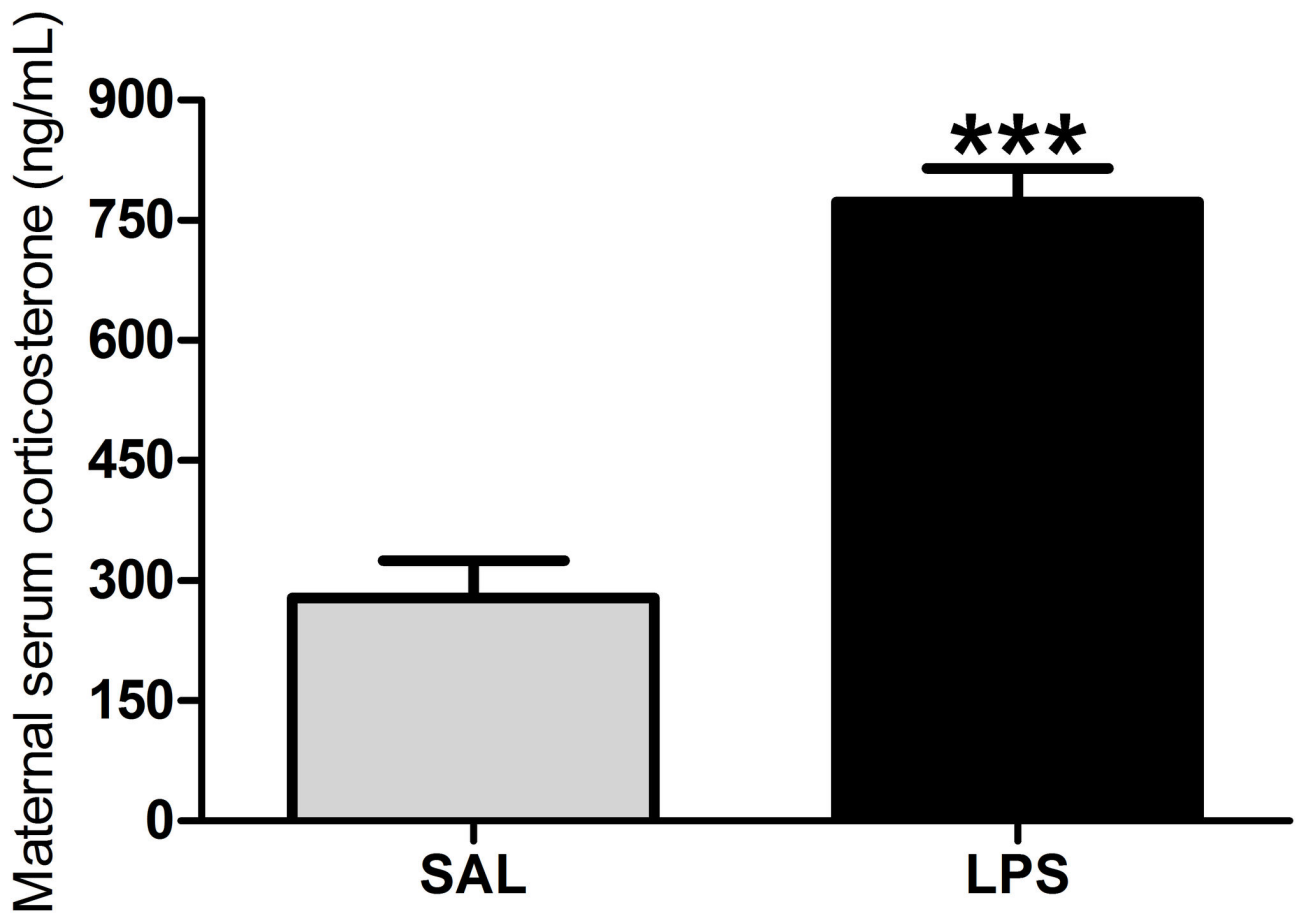
Maternal serum corticosterone levels. Effects of LPS exposure (100 µg/kg on GD 9.5) on serum corticosterone levels in pregnant rats 90 minutes after the administration of LPS. Gray bar = control (SAL) group (n = 8); black bar = LPS group (n = 9). ***p < 0.0001 (Student’s t-test) compared with the SAL group. The values are given as the mean ± SEM.

LPS exposure increased serum corticosterone levels in pregnant rats 90 minutes after exposure when compared with the control group (p > 0.0001; Figure 1). As shown in Table 1, the reproductive performance of dams exposed to LPS was also altered compared with their controls. The % of post-implantation loss was higher in the LPS-treated group. In agreement with these data, the % of live fetuses was smaller in the LPS-treated group. There were no differences between the LPS and SAL groups in the % of pre-implantation loss and in the % of implantation sites.

**Table 1:** Effects of LPS exposure (100 µg/kg on GD 9.5) on the reproductive performance of pregnant rats on the GD16.

Parameter	SAL group	LPS group	*p*
% pre-implantation loss	9.25 (0.00–15.38)	10.80 (0.00–25.00)	0.6868
% post-implantation loss	2.65 (0.00–18.18)	14.49 (11.11–33.33) *	0.0438
% implantation sites	90.75 (84.62–100.00)	89.20 (75.00–100.00)	0.6868
% live fetuses	97.35 (81.82–100.00)	85.52 (66.67–88.89) *	0.0438

* *p* < 0.05 compared with SAL group (Mann-Whitney U test).

n = 6 rats/group; data are mean ± SEM

**Figure 2:**
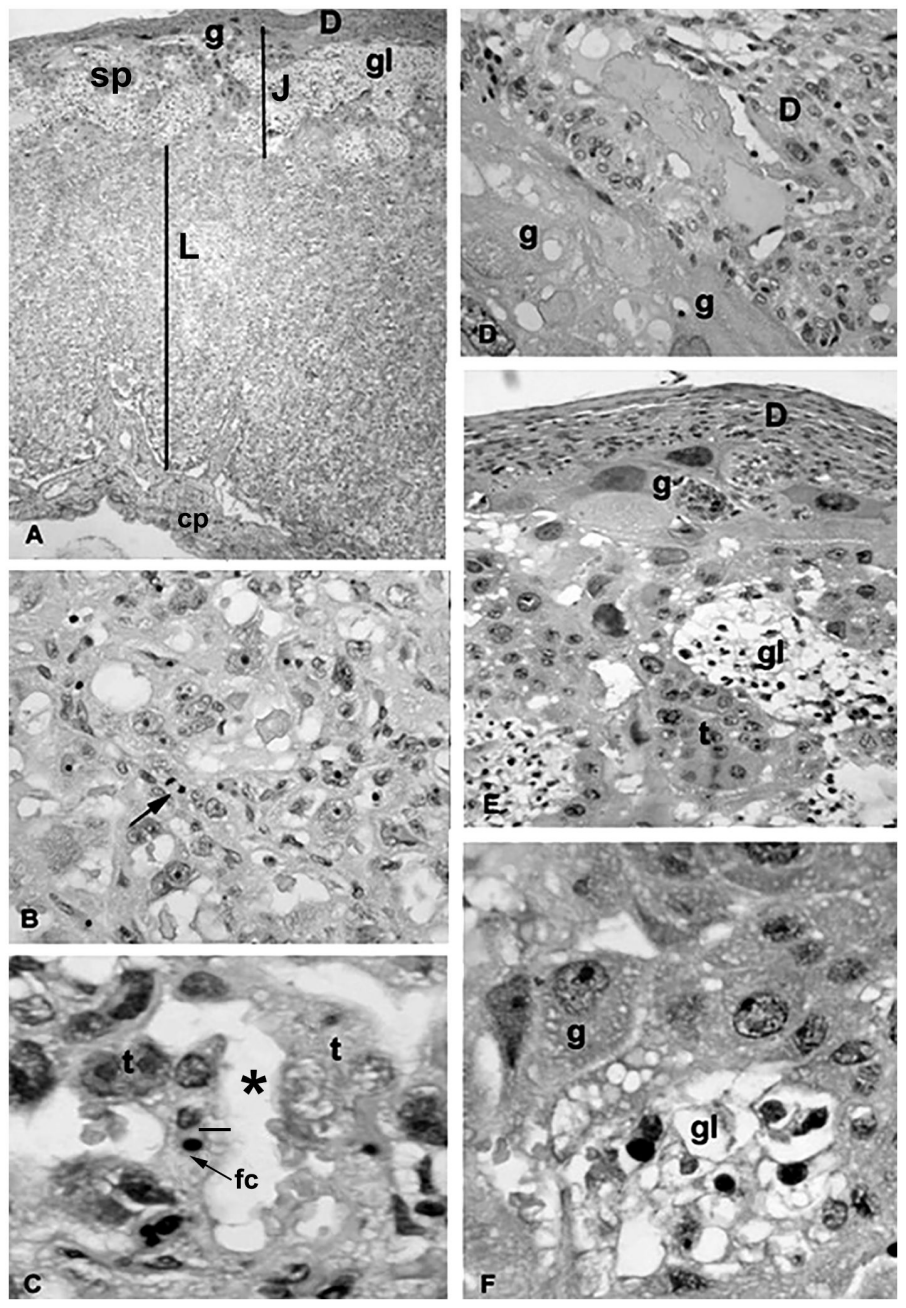
Placenta from the control group. The animals received saline on GD 9.5, and the placentas were obtained on GD 16. The photomicrographs show placental areas with normal architectures and cellular organization. A. Panoramic view showing the junctional (J) and labyrinthine (L) zones; the maternal component of the maternal-fetal interface is represented by decidua (D) and on the fetal side by trophoblast giant cells (g). Sp: spongiotrophoblast cells; cp: chorionic plate region; gl: glycogen cells. B–C. The photomicrographs show details of the labyrinthine area. Arrow in B indicates a mitotic figure; ¾: maternal-fetal barrier; *: maternal blood spaces; fc: a fetal capillary containing one erythrocyte; t: trophoblast cell layers. D–F. The photomicrographs show details of the junctional zone. In D, the border of the maternal-fetal interface shows trophoblast giant cells (g) facing the mesometrial decidua (D). In E and F, same cell types are depicted as well as glycogen cells (gl) and the remaining cells (t) of the spongiotrophoblast layer. Hematoxylin-eosin staining. A: ×100; B, D and E: ×200; C and F: ×400.

**Figure 3:**
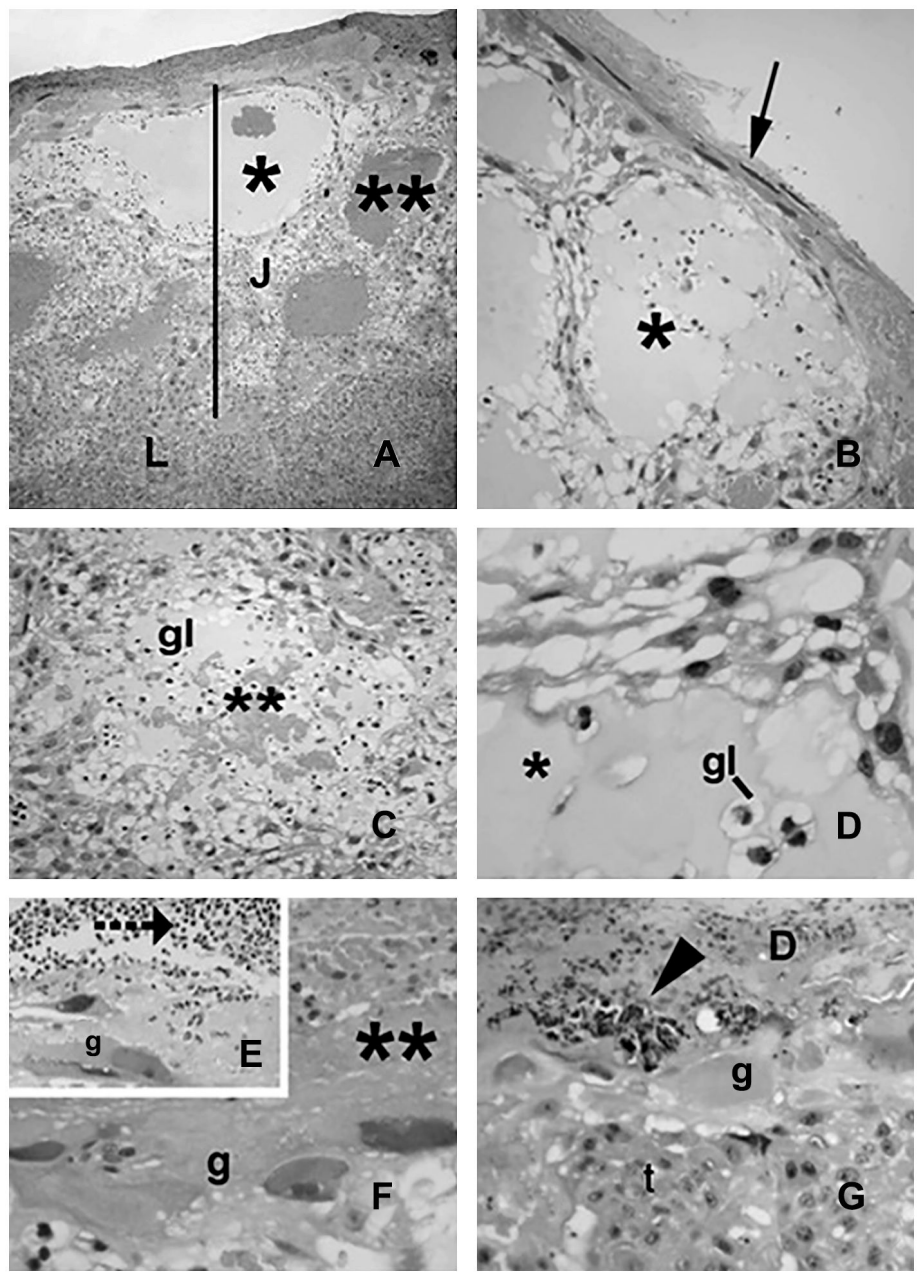
Placenta from the LPS-treated group. Effects of LPS exposure (100 µg/kg on GD 9.5) on placental tissue from pregnant rats on GD 16. A. Panoramic view showing the junctional area (J) and part of the labyrinthine (L) zone. Cavitation (cyst) inside the junctional zone is indicated by an asterisk (also depicted in Figure B and D), similar areas containing blood are identified with double asterisks (also in Figures C and F). Arrow in Figure B shows the atypical morphology of decidual cells. In E and G, note the leukocyte infiltration at the maternal-fetal interface (E, segmented arrow) and degenerative decidual cells with pyknotic nuclei (G, arrowhead). D: decidua; t: spongiotrophoblast cells; gl: glycogen cells; g: trophoblast giant cells. Photomicrographs, Hematoxylin-eosin staining. A: ×100; B–C and G: ×200; D and F: ×400.

**Figure 4:**
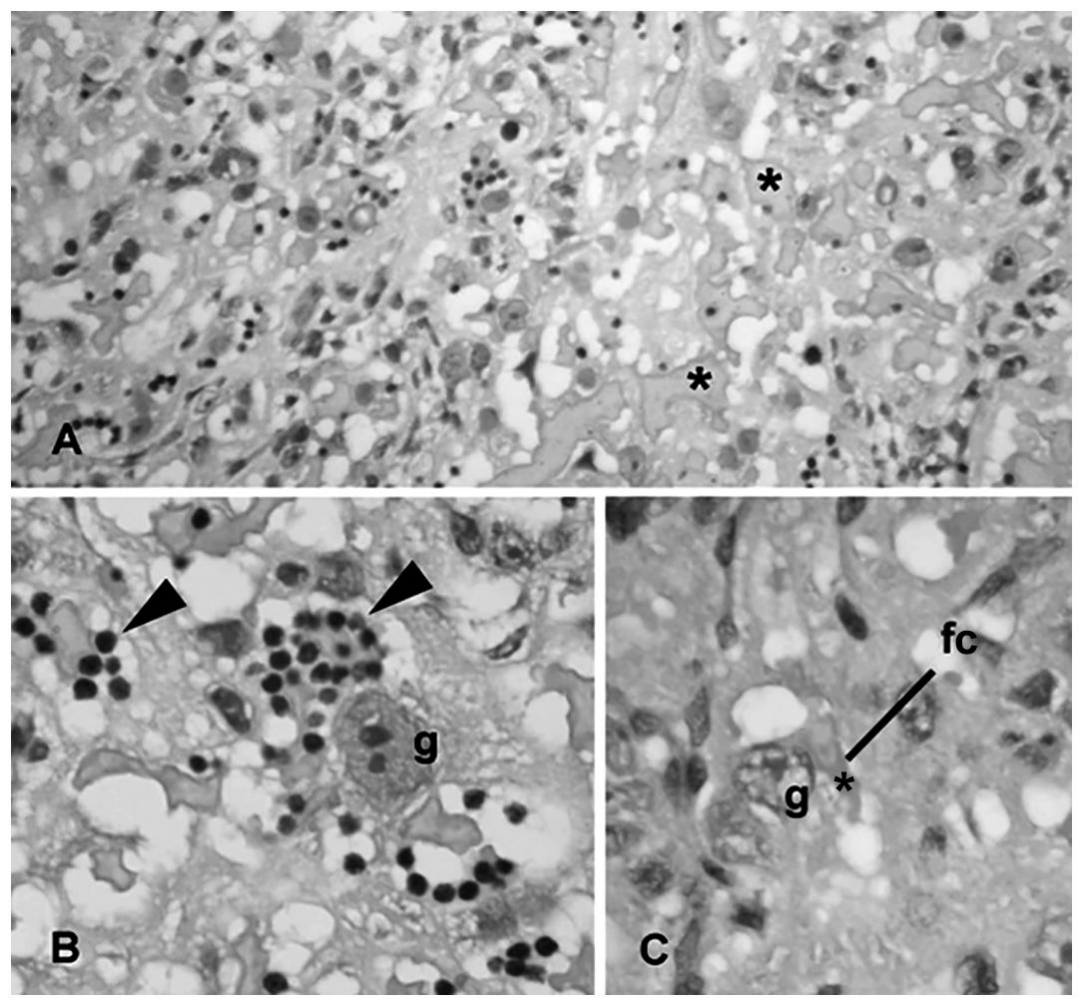
Placenta from the LPS-treated group. Effects of LPS exposure (100 µg/kg on GD 9.5) on placental tissue of pregnant rats on GD 16. A. Panoramic view showing the labyrinthine zone. Asterisks show the spaces containing maternal blood at the exchange area of the placenta. B. Note the clusters of maternal leukocytes in these spaces (arrowhead) lined by trophoblast giant cells (g) of the labyrinth. C. The photomicrograph highlights the placental barrier, clearly thickened between the spaces containing maternal blood (*) and the fetal capillary (fc). Hematoxylin-eosin staining. A: ×100; B: ×400; C: ×200.

In comparison with placentas from the control group (Figure 2), the placentas of the rats that received LPS (Figures 3 and 4) exhibited differences in the maternal (decidua) and fetal (labyrinthine and junctional zones) placental areas. These changes were more pronounced and evident in the placental junctional region (Figure 3). The hypertrophy was primarily due to the large numbers of cysts, of varying sizes, present. Some cysts were large, glassy in appearance and eosin weak-staining at the junctional zone (Figure 3). Despite this hypertrophy, the glycogen cellular compartment was clearly reduced and often represented by cellular clusters surrounding the cysts. Some of the cysts also contained extravasated blood and/or amorphous material (Figure 3). 

Trophoblast giant cells with erythrophagocytic activity were frequently observed in the placentas of the LPS-treated group. At the maternal-fetal interface, decidual cells exhibiting degenerative characteristics, including pyknotic and fragmented nuclei, were very common. These decidual cells also stained intensely with hematoxylin (Figure 3). Cells with similar characteristics were only occasionally found in the control placentas. Furthermore, at the maternal-fetal interface, hemorrhagic areas (large pockets of extravasated maternal blood) and leukocyte infiltration (mainly neutrophils) in contact with the trophoblast were specific to the treated group (Figure 3). 

The changes found in the labyrinth of the LPS-treated rats included congestion of the maternal blood spaces of the labyrinth, leukocytes infiltration and thickening of the maternal-fetal barrier, apparently caused by the hypertrophy of the trophoblast cells (Figure 4).

**Figure 5:**
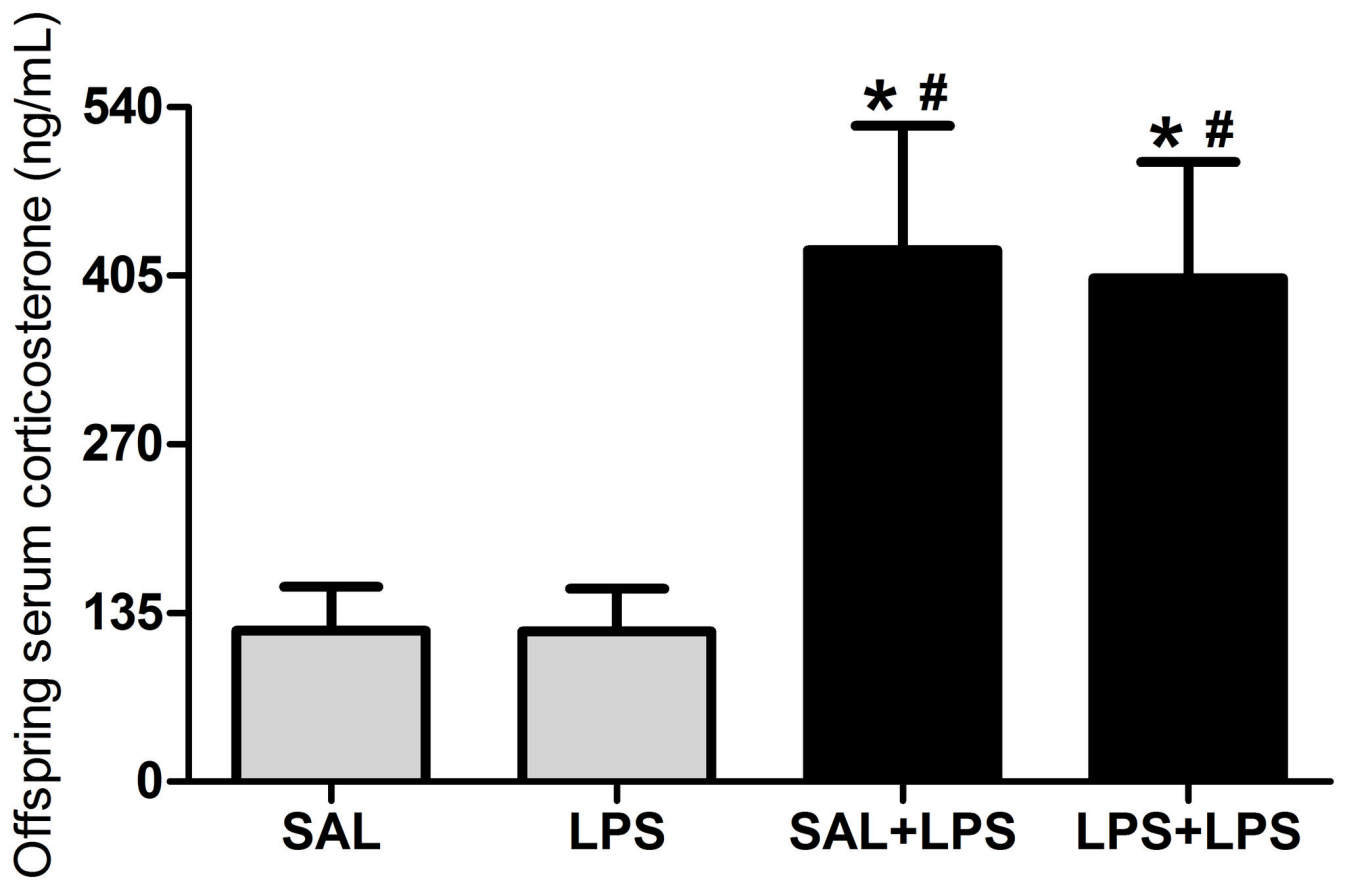
Offspring serum corticosterone levels. Effects of prenatal LPS exposure (100 µg/kg on GD 9.5) on serum corticosterone levels in adult male rat offspring on PND 60-67, with or without a LPS challenge 90 minutes prior to the test. SAL: prenatal saline injection; LPS: prenatal LPS injection; SAL+LPS: prenatal saline injection and LPS injection in the adulthood; LPS+LPS: prenatal and adult LPS injections; n = 10 rats/group. * *p* < 0.05 compared with the SAL group and # *p* < 0.05 compared with the LPS group (One-way ANOVA followed by Tukey’s test). The values are given as the mean ± SEM.

 One-way ANOVA demonstrated that prenatal LPS exposure influenced serum corticosterone levels in the offspring (F(3/36) = 5.54, p = 0.0031; Figure 5). The post-test revealed no differences between the LPS and SAL groups or between the SAL+LPS and LPS+LPS groups. However, there were differences between both groups challenged with LPS in adulthood versus both non-challenged groups, i.e., the corticosterone levels were higher in the SAL+LPS and LPS+LPS groups compared with SAL and LPS groups. This experiment revealed that prenatal LPS exposure did not interfere with the serum corticosterone levels of the offspring. The corticosterone levels increased only in response to an acute LPS challenge.

**Figure 6:**
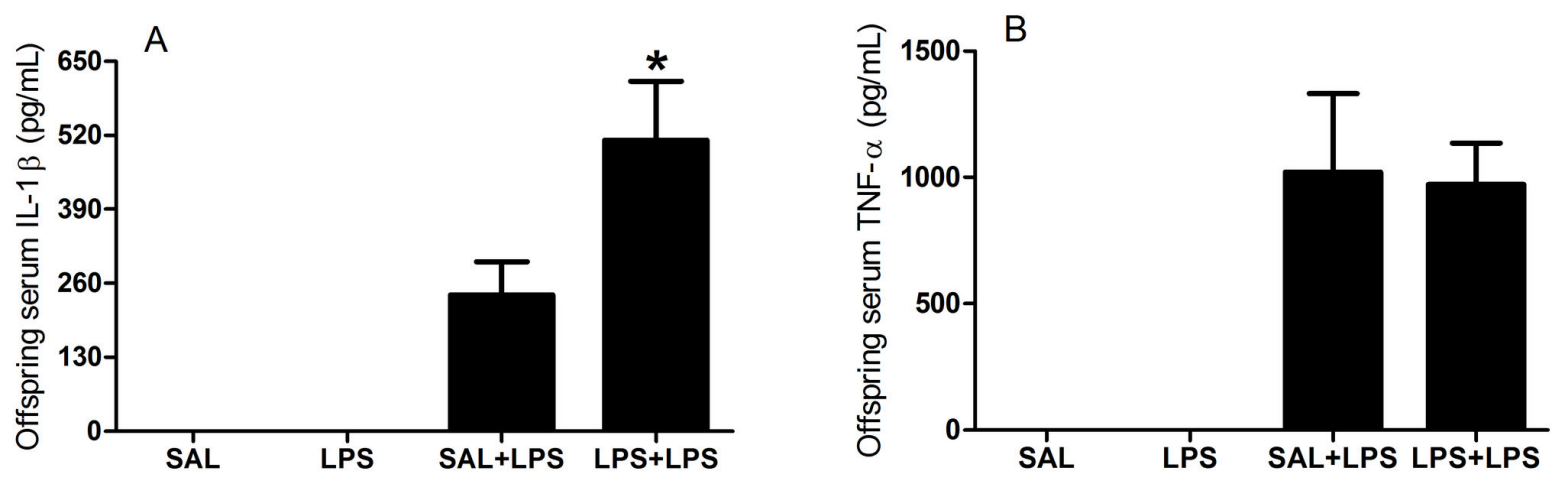
Offspring serum pro-inflammatory cytokine levels. Effects of prenatal LPS exposure (100 µg/kg on GD 9.5) on serum IL-1β and TNF-α levels in adult male rat offspring on PND 60-67, with or without a LPS challenge 90 minutes prior to the test. SAL: prenatal saline injection; LPS: prenatal LPS injection; SAL+LPS: prenatal saline injection and LPS injection in the adulthood; LPS+LPS: prenatal and adult LPS injections; n = 7-10 rats/group. Pro-inflammatory cytokines serum levels were not detected in rats that were not challenged with LPS in adulthood (SAL and LPS groups). * *p* < 0.05 (Student’s t-test) compared with the SAL+LPS group. The values are given as the mean ± SEM.

IL-1β and TNF-α serum levels were not detected in rats treated with saline or LPS during pregnancy that were not challenged further in adulthood. Thus, we applied the Student’s t-test to compare data from SAL+LPS and LPS+LPS groups. IL-1β levels were increased in rats that were prenatally exposed to LPS and subsequently challenged with LPS in adulthood (LPS+LPS) compared to the rats that were prenatally exposed to saline and subsequently challenged with LPS in adulthood (SAL+LPS, t = 2.29, df = 12, p = 0.0406; Figure 6A). The TNF-α levels did not change among the SAL+LPS and LPS+LPS groups (t = 0.14, df = 12, p = 0.8911; Figure 6B). In summary, these data revealed that prenatal LPS exposure induced a long-term effect, increasing IL-1β serum levels in adults in response to immune challenge.

## Discussion

The present results showed that LPS exposure on GD 9.5 clearly impaired the gestation of rats. Higher post-implantation loss and fewer live fetuses occurred after LPS exposure. Taken together with our previous findings of reduced the number of pups born alive, prenatal LPS significantly influenced post-implantation embryonic development. However, it not interfered with the gestational length and in the individual weights of the infantile, juvenile and adult offspring [8]. We demonstrated a toxic effect of prenatal LPS administration, significantly reducing the viability of offspring. The mechanism of the prenatal LPS inducing embryonic resorption, abortion, intra-uterine fetal death and preterm labor appears to be an indirect toxic effect resulting from the increased production and release of proinflammatory cytokines [19,20].

The implantation sites and pre-implantation loss were not affected by prenatal LPS. This result was expected, because LPS was given on GD 9.5 and the implantation of rats occurs on GD 4.5 [21]. Thus, the implantation had already occurred when the rats received LPS.

Although the placenta has protective functions for the fetus, as with any other lipid barrier, it allows the penetration of apolar substances. Furthermore, this organ contains an active transport system and facilitated diffusion, which can allow the passage of desirable, but sometimes not, molecules through specific mechanisms [22]. However, the degree of fetal intoxication and injury may be much more extreme once detoxication in the fetus is reduced due to incomplete immune system development and the scarce repertoire of biotransformation enzymes [23]. 

In addition, the current data showed placental injuries, corroborating the LPS effects on GD 9.5 and gestational impairment in rats. Analysis of placental morphology is a valid methodology to understand the effects of exposure to endotoxin in pregnancy because, in both humans and rodents, fetal placental cells are in close contact with maternal blood (hemochorial placenta) [22]. This similarity between human and rodent placentas makes it possible to speculate about how observations made in rodent models may be relevant to pregnant women.

The hemorrhagic areas, degenerative processes and cyst formation found in decidual cells, labyrinth and junctional zones characterized the toxic effects of LPS in the placenta, which surely lead to consequences for the fetuses [24,25]. The proinflammatory cytokines released by the maternal immune system in response to LPS exposure appears to be the main factor associated with these injuries. Cytokines are known to change the environmental conditions as well as the fate and function of placental cells [26,27]. Moreover, many cytokines can cross the placental barrier and induce changes in various maternal and fetal tissues [2,17]. The ability of unbalanced cytokine levels to impair placental function and commit gestational success has been thoroughly explored in the literature. For instance, exacerbated cytokine production by decidual macrophages may be involved in preterm delivery pathogenesis [28]. Here, we examined the levels of the pro-inflammatory cytokines under challenge and unchallenged conditions. Increases in these cytokines correlated with placental injury are consistent with previous findings [27,29,30].

The effects of IL-1β on the cellular and functional architecture of the developing placenta may result from several direct and/or indirect factors. Nuclear factor kappa B (NF-κB) activation by IL-1β and consequent prostaglandin synthesis, as well as expression of the type II phospholipase A2 and the PG endoperoxide H synthase (cyclooxygenase) [31], could be a plausible explanation for the edema and cavitation found in the spongiotrophoblast, for instance. Cyclooxygenase (Cox)-2 also increased in the decidua of rats exposed to LPS during gestation [32] and, as inducer of eicosanoid production, may be involved in gestational impairment [33].

The role of decidual leukocytes in the maintenance of pregnancy is not completely understood. In the present study, leukocyte infiltration increased, suggesting inflammation and possibly activation, of the maternal immune system. How much leukocyte infiltration can interfere with cell biology at the maternal-fetal interface has yet to be determined.

The current results demonstrate that exposure to LPS induced placental injury in rats. These injuries certainly contributed to the adverse reproductive events and to the previously reported behavioral and brain impairments in the offspring, including autistic-like behavior [8-10,34].

The reproductive performance and placental changes after LPS exposure may be a consequence, at least in part, to the increased levels of maternal serum corticosterone, which means that HPA axis activity also increased [13]. LPS induces pro-inflammatory cytokine release, which induces HPA axis activation [35]. Thus, in addition to the pro-inflammatory cytokines, corticosterone may also be responsible for the reproductive and placental impairments. In fact, prenatal LPS induces increased cytokine and glucocorticoids levels in maternal circulation, the placenta and fetal brain [17,27,36].

On the other hand, the previously reported behavioral and brain impairments in the offspring [8-10,34] do not appear to be consequences of changes in the HPA axis of the offspring. The HPA axis in offspring is apparently intact because prenatal LPS exposure did not affect serum corticosterone levels in adult offspring rats, although an acute response to LPS was observed in adulthood. The likely reason for the absence of HPA axis changes is that the HPA axis develops between GDs 15 and 21 [37], which is at least five days after prenatal LPS exposure (on GD 9.5). Thus, glucocorticoids and the HPA axis most likely do not impair the brain development in utero and are not responsible for impairments in the offspring.

The increased IL-1β serum levels revealed that prenatal LPS exposure modified the immune response to a LPS challenge in adulthood. We suggested this because the control group that received the LPS challenge in adulthood presented smaller IL-1β levels than the group that was prenatally exposed to LPS (LPS+LPS group), i.e., it was not just an acute response to the adult challenge, but a response to the prenatal LPS exposure. Thus, prenatal LPS exposure may have changed the immune system programming during fetal period. The adult LPS challenge that revealed the immune system changes in rats treated prenatally with LPS can provide insights into the consequences of the bacterial infections that we are constantly subjected throughout life, for example, after eating spoiled food. In fact, several studies have reported that initial exposure to prenatal environmental insult, such as infection, can render the offspring more vulnerable to the pathological effects of a second postnatal stimulus [38]. This hypothesis is called the “two hit hypothesis” and has been related to the genesis of neuropsychiatric disorders [39,40].

The increased serum IL-1β levels in adult offspring prenatally exposed to LPS corroborate with our model of prenatal LPS exposure is an experimental autism model. In addition to the previous findings of autistic-like behaviors, including impairments in communication and socialization and induction of repetitive/restricted behavior in male, but not female, rats [8,9], higher levels of IL-1β are also associated with autism. Several autistic patients presented increased serum and brain IL-1β levels [41-43]. Together with our previous studies, our model induced autistic-like behavioral and immune disturbances in childhood and adulthood, indicating that it is a robust rat model of autism.

The fact that IL-1β and TNF-α serum levels were not detected in rats that were not challenged with LPS in adulthood shows that the inflammatory process induced after prenatal LPS was, as expected, transient and did not result in permanent infectious/inflammation in adulthood. The immune activation occurred only after a new immune challenge. Our previous findings did not reveal permanent neuroinﬂammation in the striatum or the olfactory bulb (astrocytes and microglia), which agrees with our findings that prenatal LPS does not induce permanent inflammation [9,34].

Note that serum IL-1β elevation after immune challenge can be interpreted as a beneficial adaptation of the organism because increases in the production of pro-inflammatory cytokines induce sickness behavior. Sickness behavior includes immune and behavioral strategies orchestrated to combat the invading microorganism and fast healing, as well as reducing exposure of the sick animal to predation and contamination of their colony [44,45]. Thus, taking this point into account, we can state that prenatal LPS induced an improvement in the immune response of the rats. Our previous study of prenatal LPS inducing an attenuated response to experimentally induced asthma agrees with the idea of this beneficial effect [11]. However, we must take care with extrapolations because we also showed a clear toxic effect of prenatal LPS. 

In conclusion, LPS exposure on GD 9.5 increased maternal serum corticosterone levels, injured the placental tissue and impaired reproductive performance, leading to higher post-implantation loss and fewer live fetuses. In adult offspring, the HPA axis was not affected. However, prenatal LPS exposure increased IL-1β serum levels, revealing that prenatal LPS exposure modified the immune response to a LPS challenge in adulthood. Together with our previous studies, our model induced autistic-like behavioral and immune disturbances in childhood and adulthood, indicating that it is a robust rat model of autism. We hope that the present findings contribute to the understanding of autism and associated diseases.
